# Death of Dr. Hamilton

**Published:** 1886-03-20

**Authors:** 


### EDITORIALS AND MISCELLANEOUS.

THE International Medical Congress will meet in Washiugton, D. C., in September, 1887.

DEATH OF DR. HAMILTON.—We regret to learn of the recent death of Dr. D. B. Hamilton, of Minden, La. Having been for long years a subscriber to our journal, his death seems as the departure of an old friend.

PROF. H. C. WOOD, of Philadelphia, was elected president of the Association for the Protection of Scientific Research, lately organized in Philadelphia. The object is to checkmate the efforts of those who are seeking to prevent vivisection by State legislation.

IRON AND ALUM MASS.—See the advertisement of Messrs. Dicky & Anderson, on another page, who have recently assumed the management of the celebrated Iron and Alum Springs, of Virginia. This famous mass has many useful applications in medicine, and should not be overlooked by medical men in their practice.

